# Supplementary material for: Construction of a Novel Three-Dimensional PEDOT/RVC Electrode Structure for Capacitive Deionization: Testing and Performance
Source: Materials (Basel). 2017 Jul 24;10(7):847. doi: 10.3390/ma10070847 (PMC5551889; doi:10.3390/ma10070847)
Supplement: Supplementary file 1 [file materials-10-00847-s001.pdf]

Supplementary Materials: Construction of a Novel Three-Dimensional PEDOT/RVC Electrode Structure for Capacitive Deionization: Testing and Performance

Ali Aldalbahi, Mostafizur Rahaman, Periyasami Govindasami, Mohammed Almoiqli, Tariq Altalhi and Amine Mezni

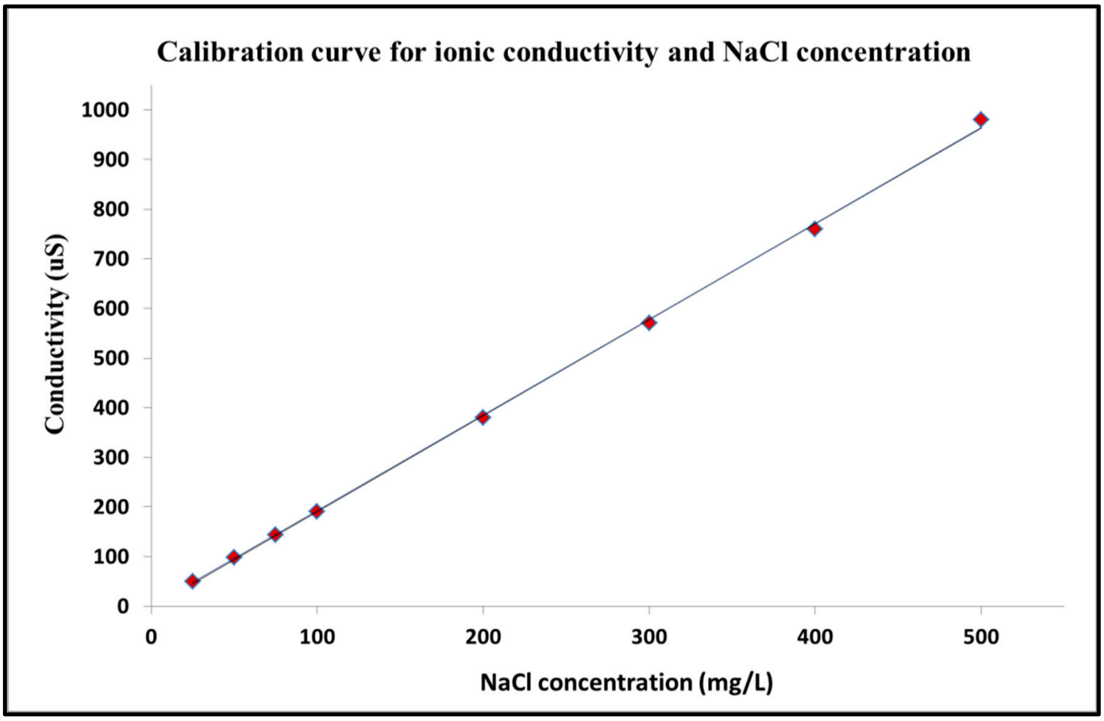

Figure S1. Calibration curve linearity for ionic conductivity *vs* NaCl concentration.

Calculation of the Ion Removal from NaCl Solution

The conductivity of the solution is measured by the conductivity meter. If the initial NaCl solution conductivity is 143.00 μS/cm and after charging the electrode it became 138.59 μS/cm, then the ion removal from the solution can be calculated. As we know from equation 1 that;

Conductivity = 1.9067 * Concentration; and hence

Concentration = Conductivity/1.9067

Therefore, Initial concentration = (143.00)/1.9067= 75.00 mg/L and

Final concentration = (138.59)/1.9067= 72.69 mg/L.

Hence, the ion removal from the NaCl solution = Initial concentration – Final concentration = 2.31 mg/L.

Calculation of Electrode Electrosorption Capacity in Terms of (mg/g) and (mg/cm^3)

If the mass of electrode is 0.05 g (having 2.16 cm^3 geometric volume and 17.88 cm^2 geometric area); volume (v) and conductivity of NaCl solution are 0.06 L and 143.00 μS/cm, respectively; and conductivity of NaCl solution after adsorption is 138.59 μS/cm, then the electrosorption capacities of the electrodes can be calculated. From the above section, we have;

Initial concentration, C_0= 75.00 mg/L and Final concentration, C_f = 72.69 mg/L.

Therefore from equation 2, we have; $M_{mass} = [(C_0 – C_f)* V] / m$

so, $M_{mass} = [(75 – 72.69) * 0.06] / 0.05 = 2.77$ mg/g

Or from equation 3, we have; $M_{volume} = [(C_0 – C_f)* V] /Z$

so, $M_{volume} = [(75 – 72.69) * 0.06] /2.16 = 0.06$ mg/cm^3

Or from equation 4, we have; $M_{area} = [(C_0 – C_f)* V] /Z$

so, $M_{area} = [(75 – 72.69) * 0.06] /17.88 = 0.008$ mg/cm^2
